# Carbon Nanostructure Embedded Novel Sensor Implementation for Detection of Aromatic Volatile Organic Compounds: An Organized Review

**DOI:** 10.1021/acsomega.2c05953

**Published:** 2023-01-23

**Authors:** Nibedita Nath, Anupam Kumar, Subhendu Chakroborty, Siba Soren, Arundhati Barik, Kaushik Pal, Fernando Gomes de Souza

**Affiliations:** † Department of Chemistry, D.S. Degree College, Laida, Sambalpur, Odisha 768214, India; ‡ Electrical and Electronics Engineering Department, IES College of Technology, Bhopal, Madhya Pradesh 462044, India; § Department of Basic Sciences, IITM, IES University, Bhopal, Madhya Pradesh 462044, India; ∥ Department of Chemistry, Ravenshaw University, Cuttack, Odisha 753003, India; ⊥ Rama Devi Women’s University, Bhubaneswar, Odisha 751007, India; # University Centre for Research and Development (UCRD), Department of Physics, Chandigarh University, Mohali, Gharuan, Punjab 140413, India; ▲ Instituto de Macromoléculas Professora Eloisa Mano, Centro de Tecnologia-Cidade Universitária, Universidade Federal de Rio de Janeiro, Rio de Janeiro 21941-617, Brazil

## Abstract

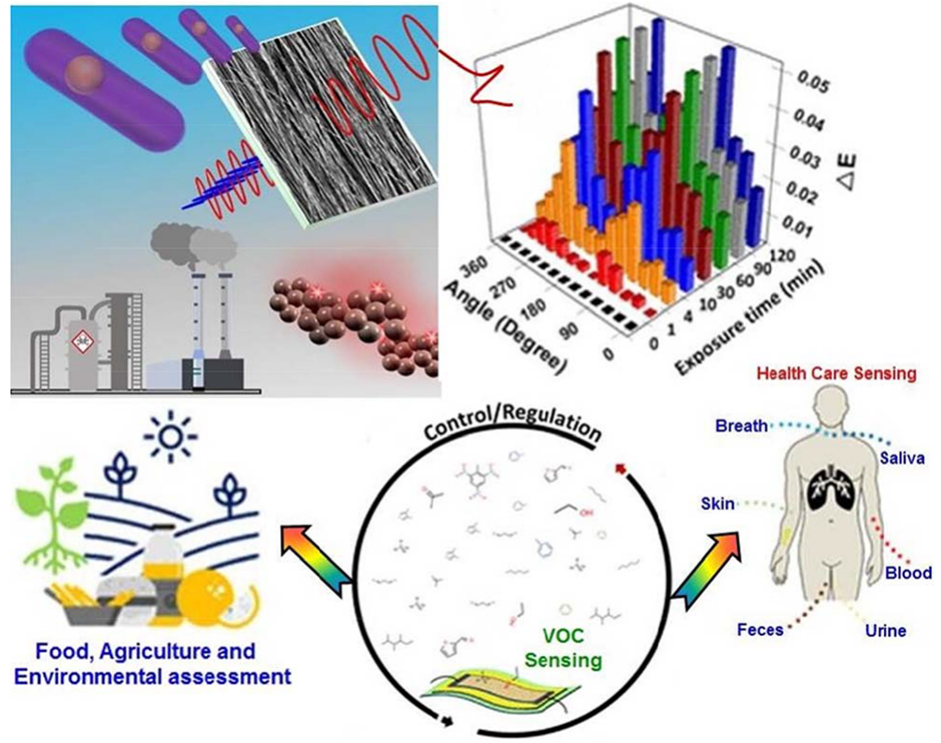

For field-like environmental gas monitoring and noninvasive illness diagnostics, effective sensing materials with exceptional sensing capabilities of sensitive, quick detection of volatile organic compounds (VOCs) are required. Carbon-based nanomaterials (CNMs), like CNTs, graphene, carbon dots (Cdots), and others, have recently drawn a lot of interest for their future application as an elevated-performance sensor for the detection of VOCs. CNMs have a greater potential for developing selective sensors that target VOCs due to their tunable chemical and surface properties. Additionally, the mechanical versatility of CNMs enables the development of novel gas sensors and places them ahead of other sensing materials for wearable applications. An overview of the latest advancements in the study of CNM-based sensors is given in this comprehensive organized review.

## 1 Introduction

Volatile organic compounds (VOCs) are substances that are evaporated and enter the environment under ordinary circumstances. They are present in a variety of products. Due to higher volatility, mobility, and resistance to degradation, VOCs can travel over large distances in the atmosphere.^1^ The most common VOCs are halogenated hydrocarbons, e.g., vinyl chloride and chloroethene, as well as aromatic hydrocarbons like: benzene, methylbenzene, xylene, and ethylbenzene.

**Figure 1:**
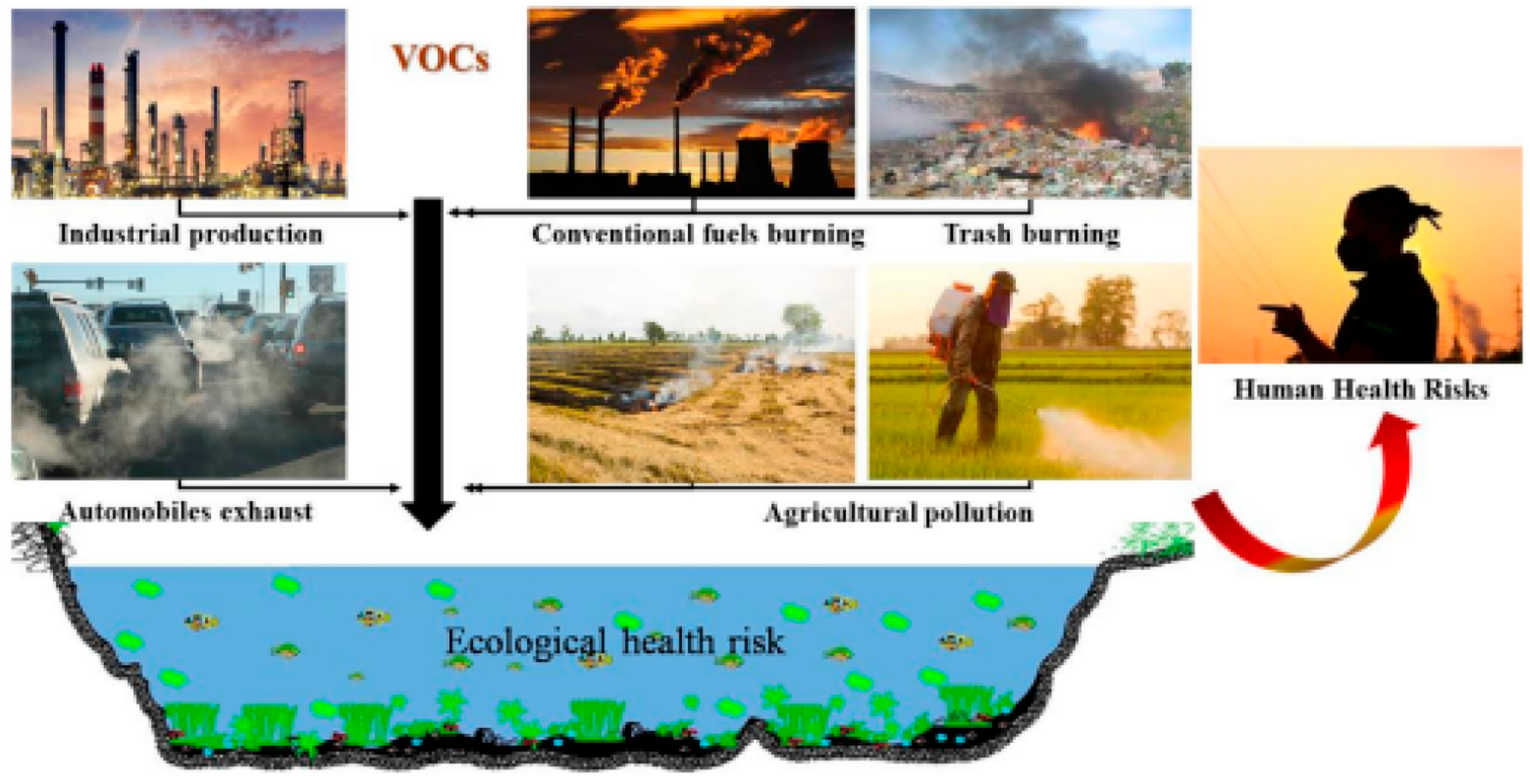
Possible origins of VOC emission. Reprinted with permission from ref (2). Copyright 2021 MDPI.

VOCs come from both natural and man-made sources. Plant emissions, naturally occurring forest fires, and anaerobic moor processes are examples of natural sources. Household and commercial processes that produce VOCs include food processing, the use of fertilizers and pesticides, septic systems, chlorination, transportation, burning hydrocarbon fuels, storing and distributing petroleum, cleaning textiles, printing, the pharmaceutical industry, and more^2^ (Figure 1).

Even though VOCs are utilized in many aspects of daily life, their exposure is dangerous. Ocular and sore throats, vomiting, nausea, headaches, loss of coordination, skin allergies, and other symptoms are brought on by small-scale exposure to VOCs.^3−5^ VOCs have been linked to long-term effects in humans, including harm to the central nervous system, reproductive system, lungs, liver, and kidneys.^6^ It is generally recognized that some VOCs might cause cancer.^7,8^ All living things frequently die when exposed to VOCs over an extended period. The primary causes of global warming are several VOCs, including methane. Additionally, certain VOCs are involved in the destruction of the stratospheric ozone.^9^

Significant approaches have been developed to discover VOC analytes in difficult matrices. Gas chromatography–mass spectrometry (GC-MS) is one of the most prominent analytical techniques for identifying VOCs.^10,11^ This is because of its consistent accuracy and specificity. Real-time monitoring and analysis of VOC gas have proven difficult due to its high cost, large equipment, and time-consuming sample preconcentration.^12−14^ According to their obvious benefits of being portable, affordable, and incredibly sensitive, gas sensors have opened new opportunities for VOC detection in relation to the aforementioned difficulties. This enables the reliability of online analysis and real-time monitoring of VOC analytes. Organic semiconductors, metal oxides, noble metals, sulfides, and carbon-based materials can be used to make functional VOC gas sensors. Carbon materials, particularly those with intrinsic nanoscale characteristics, have been identified as one of the most promising options for detecting VOC gas.^15−18^

Nowadays, carbon-based nanomaterials are encouraging the scientific community to make powerful sensor devices due to their characteristic structure, especially graphene and its derivatives such as graphene oxide (GO), CNTs, reduced graphene oxide (rGO), Cdots, and MXene. Several ways of interacting with VOCs, such as π–π stacking, electrostatic forces, and noncovalent bonding, make them suitable candidates for adsorbents.^19^ Carbon-based nanomaterials (CNMs) are appealing prospects for the future of automated sensor technology because of their excellent sensing detectability and intriguing transduction capabilities. A majority of atoms in 0–2-dimensional CNMs are exposed to the outside environment due to their huge specific surface area and remarkable sensitivity.^20−22^ Low-dimensional CNMs like CNTs, graphene, and MXene have a large specific surface area to reach a high detection sensitivity for VOCs; however, the majority of their atoms are discharged into the air.^23,24^ We focused on the most recent developments in CNMs for VOC sensing in this comprehensive review.

## 2 General Overview of the Shape and Size of rGO, CNTs, and CDs

**Figure 2:**
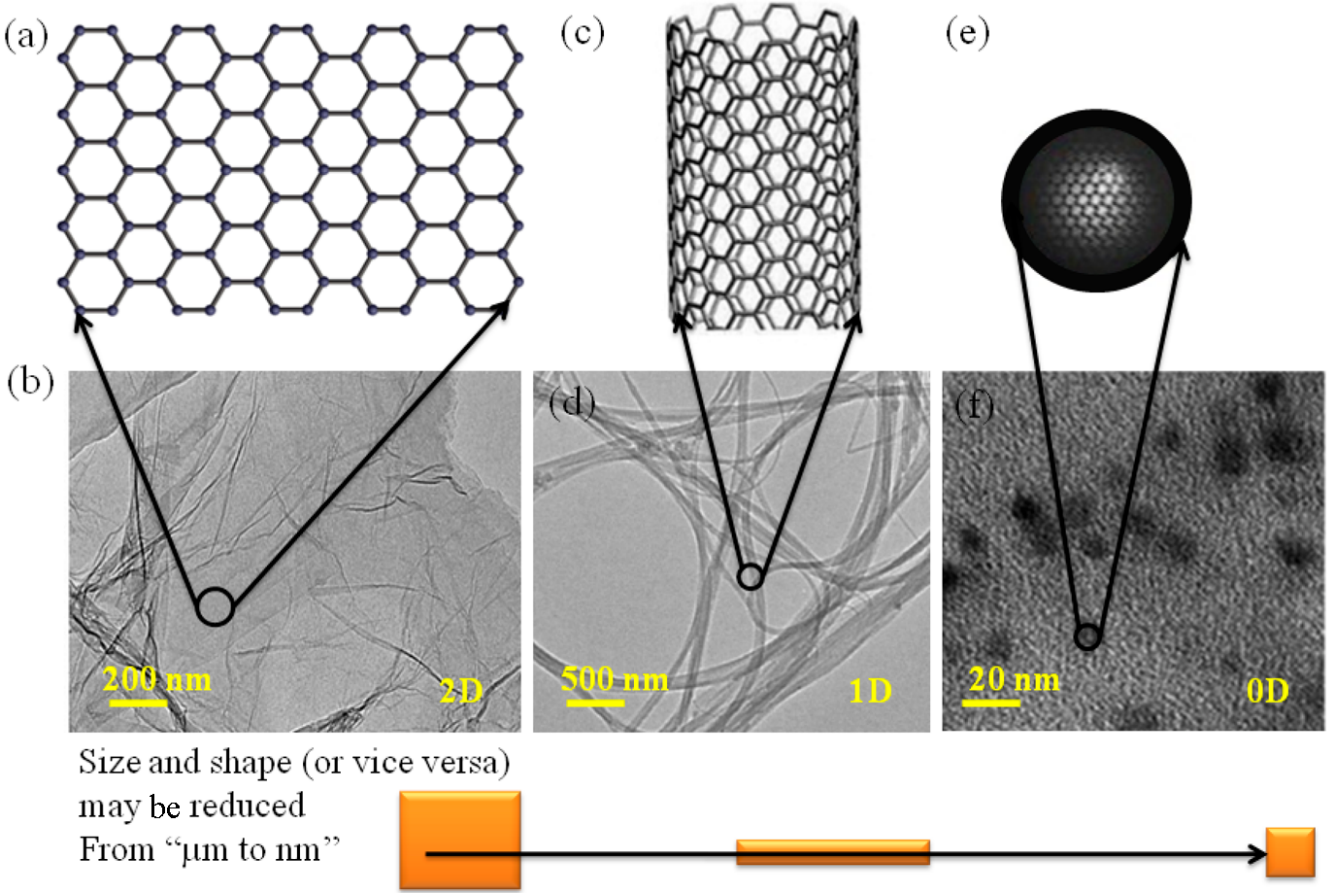
Graphical representations of TEM images of (a,b) graphene, (c,d) CNTs, and (e,f) Cdots. Reprinted with permission from refs (32), (33), and (34). Copyright 2014 Springer Nature.

The carbon nanomaterials can be synthesized with controlled shape and size from 0D to 2D, whereas 3D carbon materials are considered as graphite, diamond, etc. Except for graphite and diamond, we have focused on graphene, CNTs, CDs, and other carbon-based nanomaterials. When we talk about nanomaterials, their shapes and sizes are more critical because of surface catalytic reactions. The reduced graphene (rGO) obtained by heating or using a reducing agent on GO sheets is not spherical. Its nominal effective diameter can be calculated instead of the actual size using dynamic light-scattering (DLS) techniques. Through this technique, different scientists measured the average sizes of rGO sheet combinations of honeycomb structures of a carbon atom, which were found to be 2.93 μm, 0.5–5 μm, etc.^25,26^ The CNT, which is a circular tube-like structure due to its rolled up graphene sheets, can be a single-walled or multi-walled CNT. Hence, it is considered a 1D carbon material; its length can be extended to some micrometers long, whereas its diameter can be found to be in the nm range.^27,28^ Carbon dots (CDs) are gaining much more attention because of their biocompatibility, excellent physicochemistry, and tunable photoluminescence and are being explored as biosensors.^29,30^ They are a new type of carbon nanostructure of >10 nm size and the most water-soluble carbon material.^31^ The effective visualization of their shape and size can be studied by a TEM image, which has been shown in Figure 2.

The other derivatives or oxidized forms of graphene by oxygen functional groups such as carboxyl (−C=O), epoxy (−C–O–C−), and hydroxyl (−OH) make graphene oxide suspended in water and other polar media.^35^ This degree of oxidation is responsible for the electrical conductivity of modified graphene and the degree of oxidation depending on various synthetic routes. The carbon atoms of modified oxidized graphene are partially sp^3^ hybridized and can move above and below the plane.^36^ Another derivative of graphene-like sheets can be obtained by reducing graphene oxide through chemical reduction or simple heating methods. Park and Ruoff examined the elemental analysis of carbon and oxygen atoms (atomic C/O ratio, ∼10) for rGO made by combustion techniques and discovered that rGO is not the same as pure graphene since it contains a considerable quantity of oxygen. Furthermore, when cooled to lower temperatures, its conductivity reduces by 3 orders of magnitude, causing it to display nonmetallic behavior.^37^ In this way, a very clear difference can be seen between pristine graphene and graphene derivatives such as GO and rGO. Both pristine graphene and its derivatives are two-dimensional structures. Still, pristine graphene is relatively inefficient for the adsorption of VOC molecules. In contrast, its derivatives have more adsorption sites due to the presence of different oxygen groups and hence offer high sensitivity toward VOC molecules of the film.^38^ On the other hand, the current–voltage (*I*–*V*) characteristics for both GO and rGO exhibit ohmic contact, which is suitable for gas-sensing applications.^39^

## 3 sp^2^-Hybridized Electronic Behavior for Sensing

It is very much essential to note the origin of electrical conductivity properties of graphene, CNTs, and Cdots for sensing properties. It will help the understanding of surface interaction as a chemisorption reaction which changes the electrical properties of carbon-based materials. The 2D graphene sheet shows high carrier mobility due to planar sp^2^-hybridized atomic orbitals, and as a result, reduced graphene oxide (rGO) became a conductive active material for molecular sensors. The rolling form of the graphene sheet (i.e., a carbon nanotube, CNT) results in π confinement due to σ–π rehybridization at its circular curvature structure and creates an asymmetric distribution of σ-bonds and π-orbitals on both sides (inside and outside) of the nanotube, forming a high π-electron conjugation outside the nanotube.^40^ Thus, the surface of CNTs can donate or withdraw electrons from any electron-donating or electron-withdrawing entity during molecular interaction, thereby modulating overall electrical resistance.^41,42^ Several groups have approached the molecular orbital (MO) theory for the electronic structure of CQDs, which demonstrated that transitions have occurred in n → π* and π → π* as available transition energies.^43−45^ According to Larson and Hu, the aromatic sp^2^-hybridized carbons are responsible for their π-states. The presence of π-electron conjugation results in high charge transfer in carbon-based materials.^46^ Generally, the detection of VOCs is governed by their interaction with carbon-based materials by diffusion and sensed by the active center of carbon-based materials,^47−50^ microgravimetric sensors,^51−55^ and resistive sensors.^56−59^ When a molecule species interacts with the sidewalls of carbon-based or any semiconductor materials during gas detection, changes in conductance^60,61^ (due to charge transfer or mobility change) or capacitance^62^ (from inherent or induced dipole moments) occur.^63−65^ The interaction between the VOCs and carbon-based compounds (rGO, CNTs, and CDs) depends strongly on the architecture and design of the composites or materials which may have a larger contact area, thus allowing greater sensitivity. The CNT has two variables, SWCNT and MWCNT. Depending upon chirality, SWCNTs can be semiconducting or metallic,^66,67^ and MWCNTs have only a metallic character and are therefore unsuitable for fabricating gas transistors.

## 4 Sensing of VOCs

Recently, point-of-care applications, various disease diagnoses, atmospheric gas tracking, and other fields have significantly increased the demand for accurate VOC gas analysis.^68−70^ Meanwhile, research into the construction of sophisticated sensors for VOC detection is expanding rapidly (Stewart et al. 2020; Das et al. 2020).^71,72^ A thorough explanation of the VOC-detecting device’s sensing process is initially required to construct a powerful VOC sensor.

Research on developing gas sensors has primarily focused on nanoparticles because of their superior physicochemical characteristics and high surface-to-volume ratio. The recent discovery of CNMs has prompted^73−75^ research toward sensing technologies. These materials have been shown to be more effective in producing higher VOC sensors.^76−78^ Three steps commonly take place in gas sensors manufactured using CNMs for VOC detection: (i) trapping of VOC gas, (ii) their interaction with the sensing active site, and (iii) dispersing of the VOC gas. Focusing on the complex interactions between the VOC gas and the active center that detects them, CNMs have been used to create a range of VOC gas sensors, including optical^76,79−81^ and microgravimetric ones.^77−86^ Regarding optical sensors, spectrometric or colorimetric variations in the sensing materials are brought on by the intermolecular interactions among VOC samples and the active center^76,79−81^ and resistance-based sensors.^87,78,88−90^

## 5 CNM-Based Sensor for VOC Detection

Due to the considerable electrochemical, mechanical, optical, and thermal properties of CNMs, many research studies have been done in this area during the last three decades. Fullerenes, carbon nanotubes, graphene, carbon dots, carbon nanohorns, and carbon black are examples of zero-, one-, two-, and three-dimensional CNMs that have shown such inherent properties that can be easily modified in the development of cutting-edge innovation for sensing applications. Nanomaterials have opened up new pathways and options for sensing analysts or target molecules.^91^

Carbon-based nanostructures have several benefits over other commonly used sensor materials, including simple manufacturing procedures, unexpected physiochemical characteristics, greater sensing properties, environmentally friendly materials, improved detection accuracy, high flexibility, sensitivity, and reliability. They also have a large surface-to-volume ratio, high stability, high electrical conductivity, biocompatibility, reduced size, and an improved surface area. CNMs are being studied as a result of further utilization of efficient sensor technology.^92,93^

Carbon nanostructures like CNTs and graphene can be used to detect extremely small levels of danger and greenhouse gases. Therefore, using the CNM-based sensor to develop susceptible, small energy gas sensors is both of significant academic interest and of enormous economic significance.^94^ The many CNM-based sensors utilized for VOC detection are listed in Table 1 in detail.

**Table 1:** Various CNM-Based Sensors for VOC Detection

CNMs	VOCs	temperature (°C)	response/recovery time (s)	ref
CNT/SnO_2_	CH_3_OH/C_2_H_5_OH	250–300	20/30	(95)
ZnO/MWCNT nanorod	C_2_H_5_OH	300	5/8	(96)
SnO_2_/MWCNT	C_3_H_6_O	RT	7/8	(97)
PMMA/POSS/CNT	HCHO	RT	<5 s	(98)
Graphene/ZnO	C_2_H_5_OH	340	5/20	(99)
Graphene/Ni-SnO_2_	C_3_H_6_O	350	5.4	(100)
Graphene/ZnO	acetone	RT	–	(101)
Graphene/ZnO	HCHO	RT	36	(102)
rGO/SnO_2_ aerogel	C_6_H_6_O	RT	2.43/1.06	(103)
rGO/α-Fe_2_O_3_ nanofiber	C_3_H_6_O	375	3/9	(104)
rGO/ZnSnO_3_	HCHO	103	31/–	(105)
rGO/DF-PDI	TEA	RT	64/128	(106)
ZnO quantum dot/graphene	HCHO	RT	30/40	(107)
Au@NGQDs/TiO_2_	HCHO	150	18/20	(108)
3D Mxene framework	acetone	RT	1.5 min/1.7 min	(109)
Ti_3_C_2_T_*x*_	C_2_H_5_OH	RT	–	(110)

## 6 CNT-Based Sensor for VOC Detection

A one-dimensional carbon allotrope known as a carbon nanotube (CNT) is composed of sp^2^ carbon atoms organized in cylindrical tubes with diameters ranging from 1 to 100 nm. Single-wall carbon nanotubes (SWCNTs) and multiwalled carbon nanotubes (MWCNTs) are the two types of CNTs used most commonly. MWCNTs, on the other hand, are composed of multiple sheets of concentric single-walled graphene cylinders with an interlayer spacing of 3.4 and are held together by van der Waals forces.^111^ SWCNTs are the rolled form of the graphene sheet. Hexagonal rings make up CNTs and control their size, curvature, and electronic properties. Chirality refers to the configuration of carbon hexagonal rings in nanotubes.^112^

Since 20 years, CNTs have been made on a massive scale for numerous uses. Arc discharge,^113^ CVD,^114^ and laser ablation^115^ are common techniques for making CNTs. The CVD process and the discharge method are more advantageous for the mass production of high-quality CNTs.^114^

One of the oldest clinical practices is breathing analysis used for medical purposes. The binding of volatile compounds to a sensor array, the creation of sensor modifications, which produce distinctive patterns of signals, and the integration of signal patterns allowing categorization are the main phases in the breathing analysis working principle, which also is based on the human olfactory system.^116^ A quick, painless, inexpensive, noninvasive method for early disease detection and ongoing physiological monitoring is breathing analysis.^116−118^ Analyzing the diaphragmatic movement when breathing can help detect human disorders like atypical sleep problems, asthma, heart attack, and lung cancer early on.^119,120^ Due to their appealing qualities, such as good biocompatibility, wearing comfort, low cost, as well as sensitivity to breathing activities in the aspect of lower frequencies and slight amplitude body motions, triboelectric nanogenerators (TENG) have recently been widely used for self-powered respiration monitoring. TENG-based respiration sensors can accurately and continuously track physiological respiratory behaviors and exhaled chemical regents for individualized health care.^121^ Further, the 'Liu' research group designed a respiration-driven triboelectric sensor (RTS) for concurrent biomechanical and biochemical breath measurement that can directly transform breath flow into electricity.^122^ The Wang research group also created an integrated triboelectric self-powered respiration sensor (TSRS) to track both human breathing patterns and the amount of NH_3_ in exhaled gases.^123^

**Figure 3:**
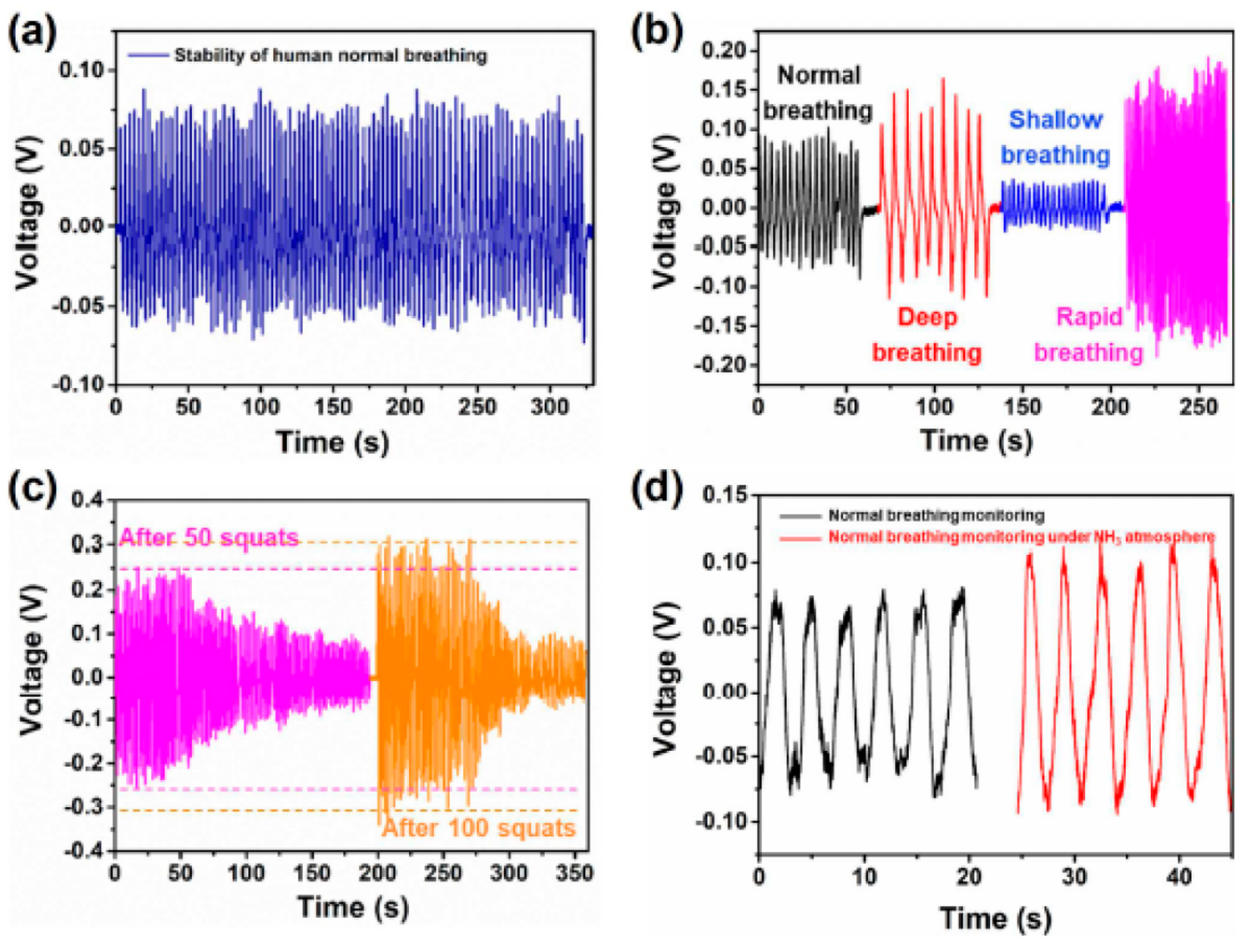
Wearable TSRSs make it possible to monitor breathing in real time. (a) The stability of TSRS driven by regular breathing in humans. (b) The TSRS’s output voltage when four distinct breathing patterns are used. (c) Active monitoring of human breathing after 50 and 100 squats, correspondingly. (d) Evaluation of output signals both with and without NH_3_ injection from exhaled breath. Reprinted with permission from ref (124). Copyright 2019 Elsevier.

'Su' and his research team created a triboelectric self-powered respiration sensor (TSRS) in 2019 to monitor human breathing patterns and the amount of NH_3_ in exhaled gases. Figure 3 shows a TSRS that is mounted to a person’s chest and tracks their breathing.^124^ Su et al. created an alveolus-inspired membrane sensor (AIMS) for self-powered wearable nitrogen dioxide detection and personal physiological evaluation in 2020. This sensor may be used to detect human breath activities for breath analysis.^125^

Using a self-validating 64-channel sensor array based on semiconducting single-walled carbon nanotubes, the Panes–Ruiz research group showed precise detection of H_2_S below breathing concentration levels in humid airflow (sc-SWCNTs). The repeatable sensor construction method is based on controlled multiplexing dielectrophoretic sc-SWCNT deposition. The sensing region is developed and produces gold nanoparticles that solve detection at room temperature by leveraging the affinity of gold and sulfur atoms in the gas. The estimated limit of detection (LOD) for sensing devices functionalized with optimum nanoparticle dispersion is 3 ppb, and their sensitivity is 0.122%/ppb. Our sensors surpass certain electrochemical sensors that seem to be available on the market in terms of improved stability and response levels despite self-validation.^126^

According to the kind and concentration of various surfactants, Chatterjee et al. investigated how several lung cancer VOCs found in human breath impacted the selectivity and sensitivity using CNT-based sensors (nonionic, cationic, and anionic). The performance of the sensors is found to be affected by both the interaction of the surfactant with the analyts and the supramolecular assembly with CNTs. Surfactant–CNT sensors were produced using the spray LbL approach. Water and other polar VOCs, particularly methanol and other alcohols, have demonstrated good sensitivity to CNT-DOC sensors. TX405-CNT sensors were sensitive to three chemicals: benzene, chloroform, and *n*-pentane. Except for isopropanol, the SDBS-CNT sensors could detect water, acetone, chloroform, and ethanol but not many biomarkers. *N*-Pentane, acetone, isoprene, and ethanol were all reactive substances for the BnzlkCl-CNT sensors. Although it was shown that pristine CNTs responded best to the majority of the aromatic VOCs in the collection, CTAB-CNT sensors were just moderately sensitive to most VOCs and lacked significant selectivity.^127^

**Figure 4:**
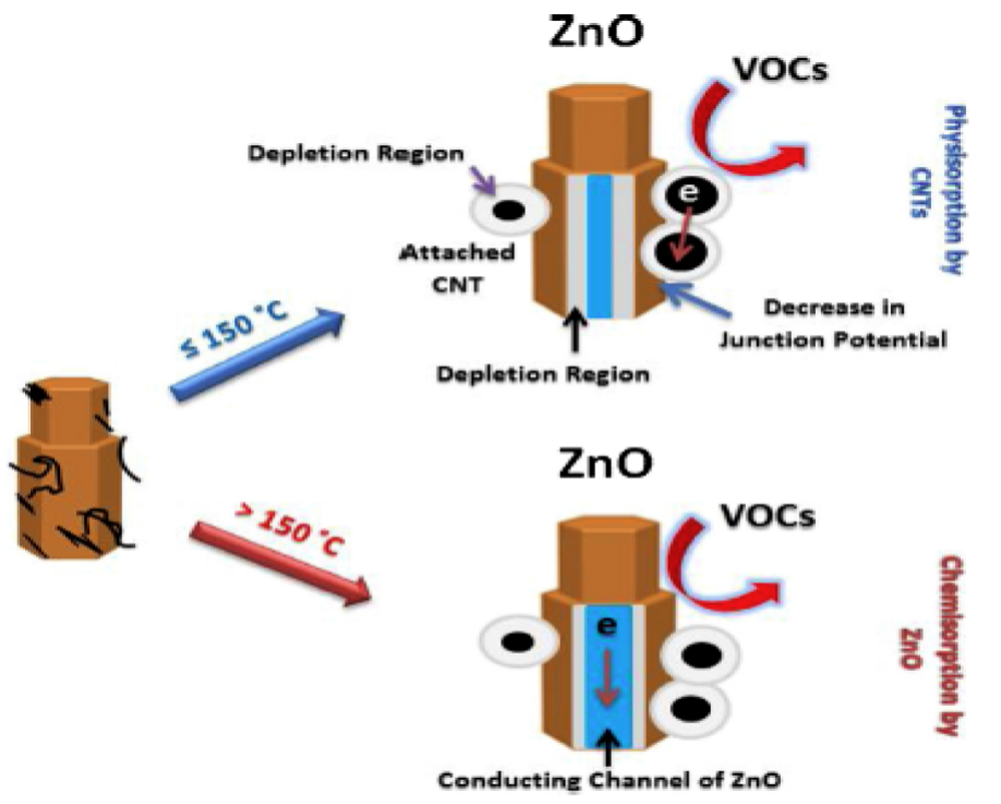
CNT/ZnO composite sensor’s VOC detection technique in the low- and high-temperature regions. Reprinted with permission from ref (129). Copyright 2021 Elsevier.

Moreover, 'Sinha' research group used zinc oxide (ZnO) and CNTs to create a composite-based chemiresistive sensor. A mechanism has been linked to the VOC adsorption process’s temperature- and material-dependent switching. Due to their simplicity in low-temperature synthesis and the large variety of VOC sensing, stability, or other advantageous qualities, ZnO/CNT composites have been selected in this work as a crucial material above other metal oxides. Additionally, it is anticipated that a p–n heterojunction will develop at every point in which CNT and ZnO are in contact. This will also impact the composite sensor’s ability to sense. A potential is created as an outcome of the interaction of n-type (ZnO) and p-type (CNT) materials. As a result, if VOCs were adsorbed onto the surface of a composite sensor, their potential should decrease.^128^ The total sensor system also benefits from this drop in junction potential. Figure 4 depicts an electron transport route schematically in connection to the phenomenon.^129^

**Figure 5:**
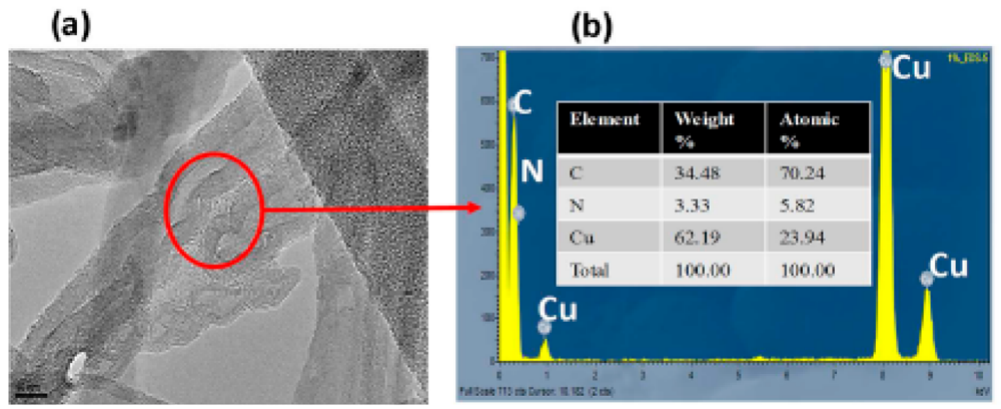
Caf-CNTj characterization: (a) high-Resolution TEM; (b) matching EDS spectra; and (inset) Caf-CNTs element table. Reprinted with permission from ref (130). Copyright 2020 Elsevier.

For the purpose of detecting volatile VOCs, the Turner research group developed vapor quantum resistive sensors (vQRSs) utilizing random networks made of carbon nanotubes (CNTs) that have been functionalized with caffeine. Energy-dispersive X-ray spectroscopy (EDS) and a localized transmission electron microscopy (TEM) image both demonstrate that caffeine has moved to the CNT–CNT junction (CNTj). Furthermore, at the CNT intersection, we conducted localized EDS (Figure 5a,b). According to the elemental analysis, about 5% of atomic nitrogen is present. Such findings prove that caffeine molecules can cluster at the CNTj of random networks and thus are noncovalently bound to CNT surfaces.

Specific VOCs like acetone, toluene, and ethanol have increased sensitivity and distinct selectivity and a quick response time with caffeine-enhanced CNT junction (Caf-CNTj) vQRSs. According to molecular dynamics simulations, the sensor’s sensitivity is diffusion-limited, with ethanol actively contributing (MD). The created Caf-CNTj sensors could be a strong contender for various activities performed by an electronic nose, including breath analysis and food quality monitoring.^130^

**Figure 6:**
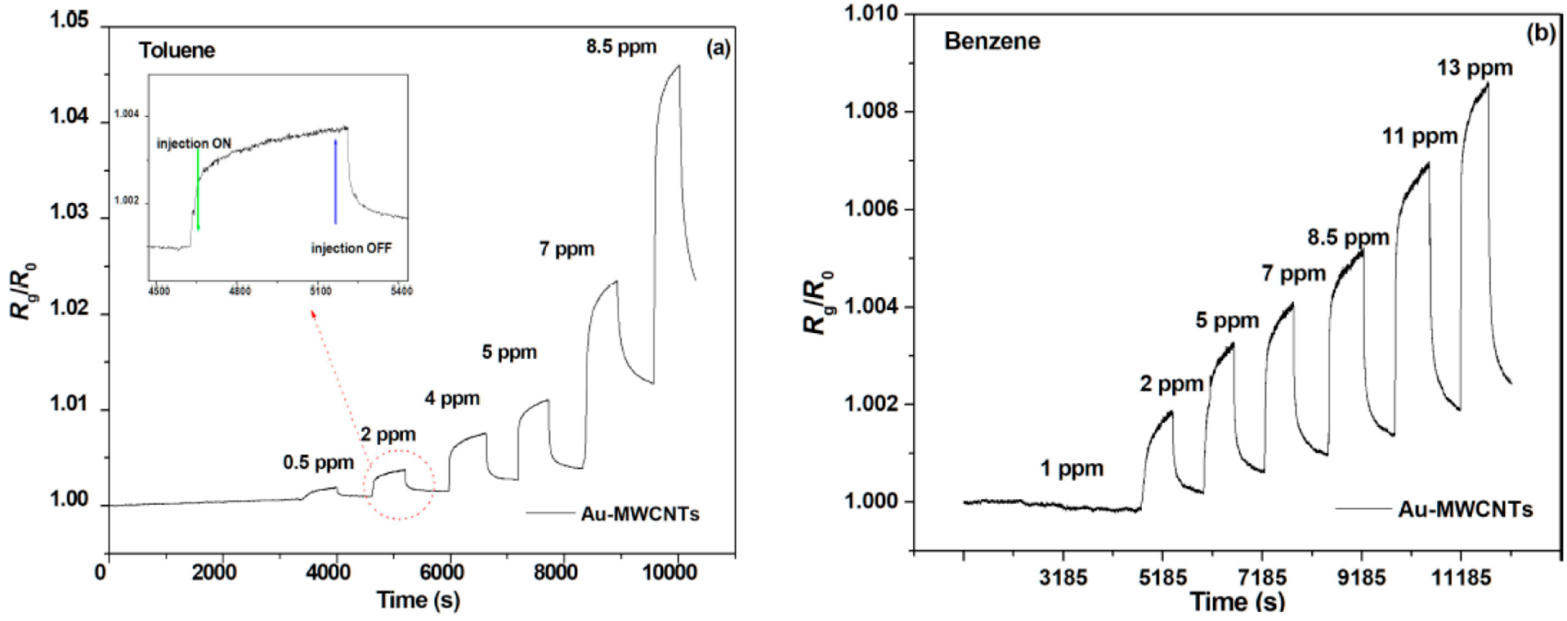
(a, b) Au-MWCNT sensor response to injections of toluene and benzene at various concentrations. Reprinted with permission from ref (131). Copyright 2019 Belestein.

Bohli et al. created a room-temperature toluene and benzene sensor in 2019 using MWCNTs functionalized with a long-chain thiol self-assembled monolayer, 1-hexadecanethiol (HDT), and decorated with Au nanoparticles. The thiol monolayer adhering to the MWCNTs was examined using FT-IR and high-resolution TEM, which were used to describe the gold nanoparticle decorating. The ability of Au-MWCNT and HDT/Au-MWCNT sensors to detect VOCs at levels as low as ppm is evidence that the self-assembled layer enhances the sensing selectivity, sensitivity by a factor of 17, and response dynamics. The response of the Au-MWCNT sensor to various vapor injection amounts for toluene and benzene at room temperature is shown in Figure 6a,b.^131^

NiWO_4_ microflowers (MFs) were used to embellish MWCNTs in composites for the detection of NH_3_. The material’s porous nature, the large specific surface area, and the p–n heterojunction formed by the MWNTs and NiWO_4_ contribute to the better sensing capabilities of this composite. The sensor based on 10% (MWN10) has the best gas sensitivity, with a sensitivity of 13.07 to 50 ppm of NH_3_ at room temperature and a detecting lower limit of 20 ppm, according to the gas sensitivity of the sensor based on daisy-like NiWO_4_/MWCNTs.^132^

## 7 Graphene-Embedded Sensor for VOC Detection

Graphene has garnered a great deal of scientific attention since Novoselov and associates used mechanical exfoliation to synthesize it for the first time in 2004,^133^ winning them the Nobel Prize for their contribution to Physics in 2010.^134^ Pure graphene and graphene oxide (GO) are the family members of “graphene”. Graphene is a single layer of graphite formed of predictable hexagonal arrangements of 2D carbon atoms. This arrangement is identical to that of graphite. Graphene is relatively light, weighing just around 0.77 mg per square meter.^135^

**Figure 7:**
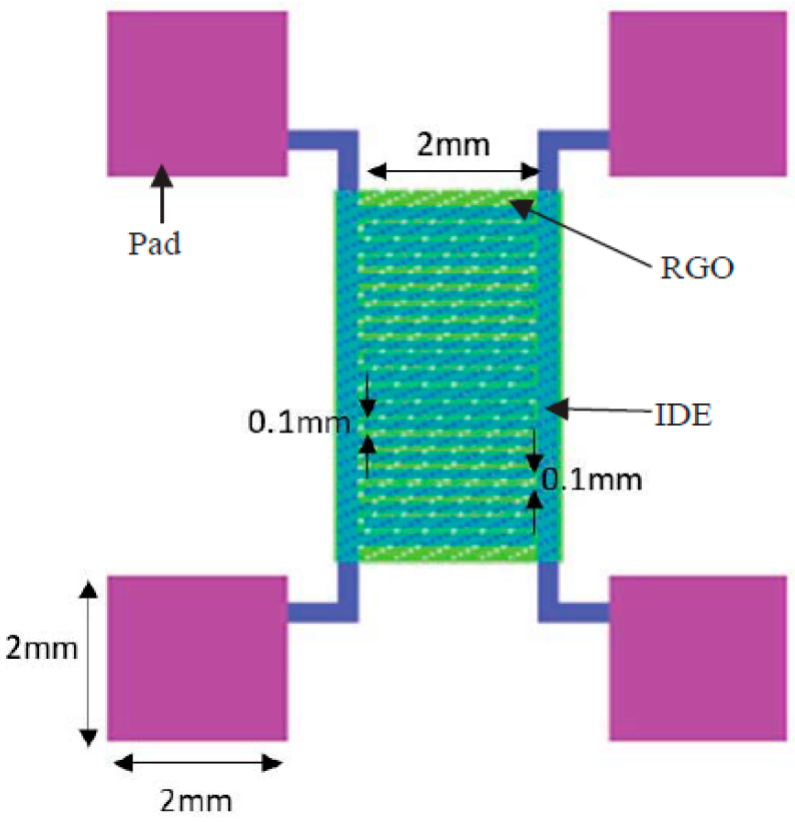
VOC sensor from the top. Reprinted with permission from ref (145). Copyright 2021 AIP Publishing.

Smart electronic material 'Graphene' has many unique properties and distinguishing characteristics. The structure’s extremely high electron mobility (200 000 cm^2^ V^1^ s^1^), much more significant than carbon nanotubes (CNTs), is made possible by its zero band gap.^136^ It possesses exceptional mechanical strength, an extremely high surface-to-volume ratio,^137,138^ high capacitance,^139,140^ outstanding thermal conductivity,^141^ exceptional electrical conductivity, and the possibility for atomically clean graphene sheets on the graphene lattice.^142^ In addition to such amazing properties, graphene also enables sensitive analyte detection because of its exceptionally low electrical noise.^143^ Functionalized reduced graphene oxide (RGO) VOC sensors for detecting the ppm level of VOCs was developed by Tombel et al. (Figure 7).^144^ Using a Ti/Pt integrated electrode and functionalized nanomaterial on RGO thin film, a single semiconductor sensor was assembled on a SiO_2_/Si substrate (IDE). The three VOCs that were the focus of the detection studies were acetone, toluene, and isoprene. These experiments were conducted at ambient temperatures of 30 °C and 40% relative humidity.^145^

Reduced graphene oxide (rGO)/metalloporphyrin-based VOC sensors are simple and affordable to make and can precisely detect VOCs linked to a number of diseases often present in human breath. They have been described by the Lee research group. Using a drop casting technique, we created an rGO-metalloporphyrin-sensing array and examined it with EDS and scanning electron microscopy (SEM). Next, three disease-related breath VOCs (acetone, ammonia, and isopropanol), as well as carbon monoxide, were introduced to the detecting array.^146^ ZnO nanowire–rGO nanocomposites are created by combining ZnO nanowires and graphene oxide (GO) in various ratios. This nanocomposite is utilized as a sensor to detect NH_3_, and it performs substantially superior to pure reduced graphene-oxide-based gas sensors, exhibiting an outstanding response (19.2%) to NH_3_ at ambient temperature.^147^

Gupta et al. investigated the use of thin coating rGO as a sensing material on a QCM sensor to detect VOC vapors at ambient temperature. To make the rGO suspension, the aqueous suspension of GO paste is first reduced with ascorbic acid. The rGO thin films were cast using the drop-cast technique and dried at room temperature. For their contaminant-free wrinkled form, multilayered graphene structure, high mobility of 2 × 10^4^ cm^2^/(V s), and low resistivity of 4 × 10^–1^ cm, acetone molecules are more readily absorbed on the surface of rGO thin films. As a result of the rGO sensing material used in the QCM gas sensor, rapid reactions are possible. The QCM gas sensors’ quick reaction and recovery periods of 20–30 s have been proven using commercially available rGO thin sensing films.^148^

**Figure 8:**
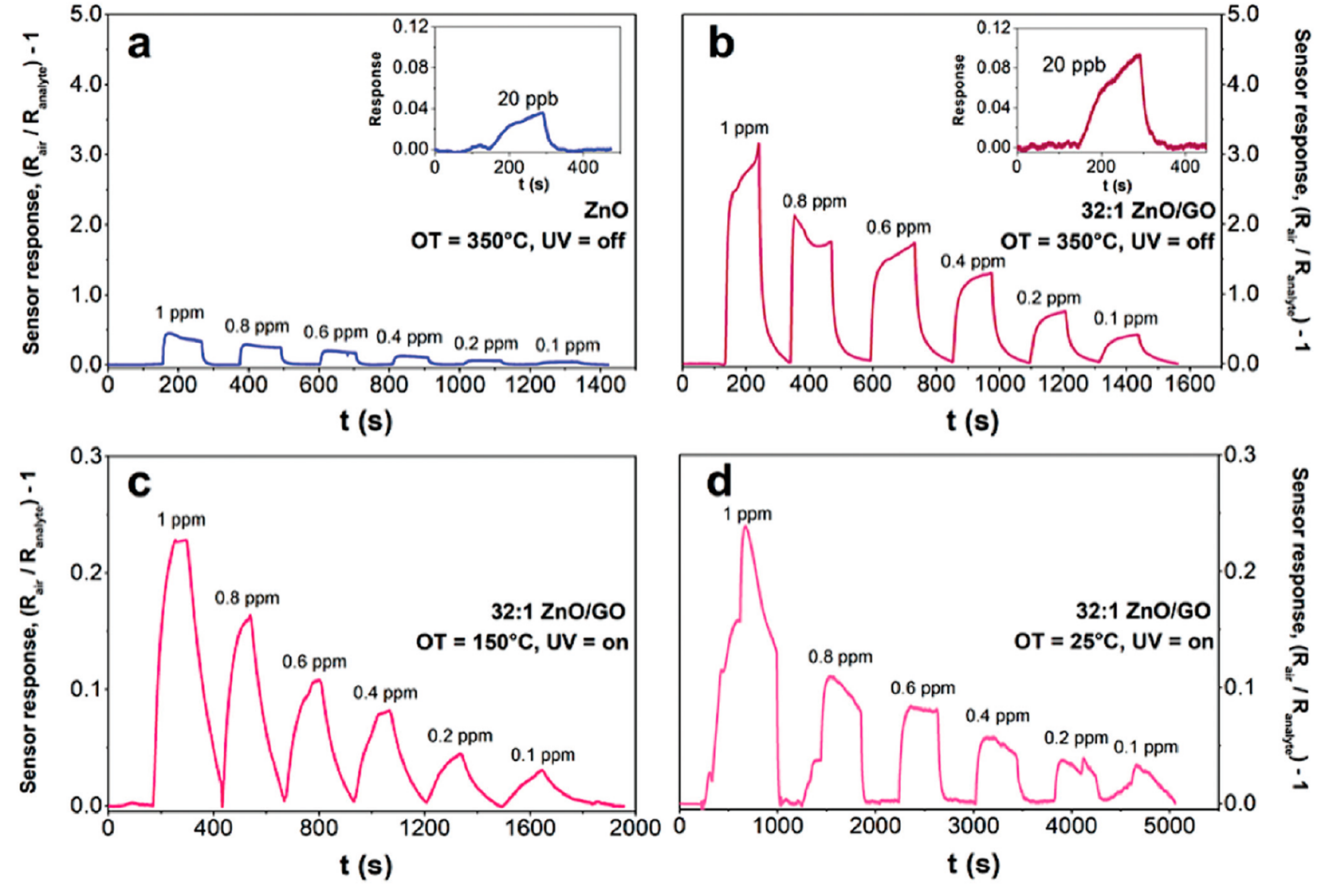
(a–d) The responses of a pure ZnO sensor and a hybrid 32:1 ZnO/GO sensor to acetone concentrations ranging from 1 ppm to 20 ppb in simulated air (20% O_2_–80% N_2_). Reprinted with permission from ref (149). Copyright 2019 RSC.

It is claimed that to produce nanostructured detectors GO nanodomains of p-type were carefully inserted into an n-type 3D ZnO nanoarchitecture. These ultraporous nanoheterojunction networks’ features were investigated using physical and chemical approaches, demonstrating how GO affects the networks’ ability to sense chemicals and light. By employing UV light activation of the sensing reactions, these nanocomposite materials were also employed to detect many common VOCs, including ethyl alcohol, propanone, and ethylbenzene, down to ambient temperature. Here, Figure 8a–d shows how exposure to UV light, temperature, and pure ZnO or 32:1 ZnO/GO films’ chemical sensing affects reactions to acetone.^149^

**Figure 9:**
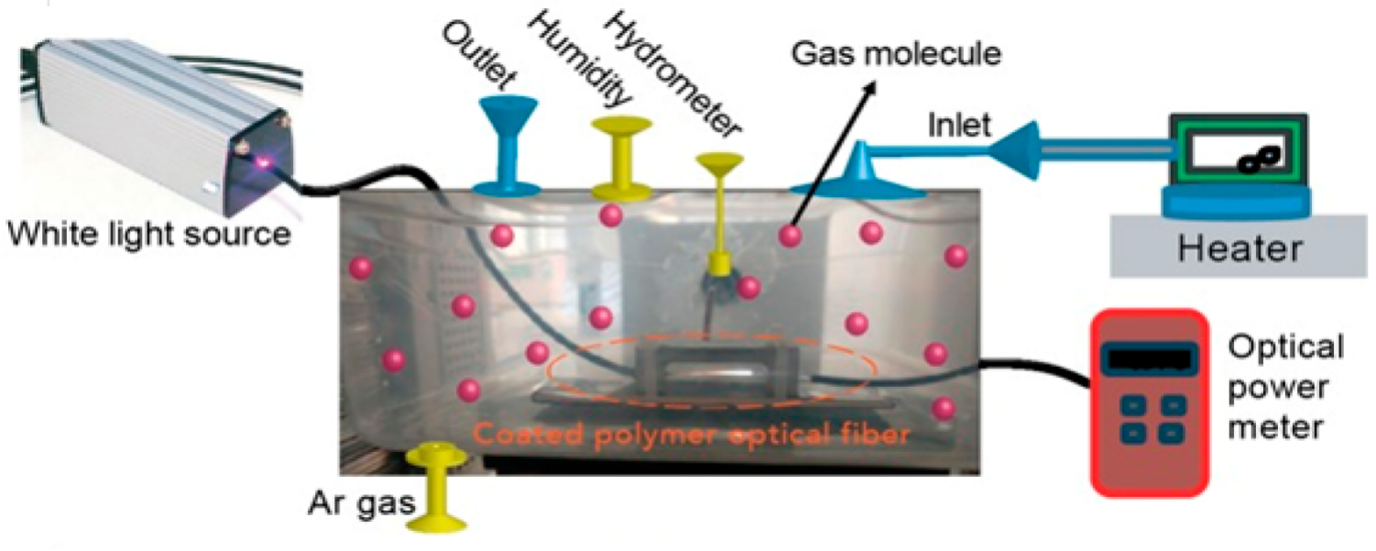
Experimental setup and potential utilization to evaluate the performance of polymer fiber optic sensor N-CNPs and N-GQD-NSs is schematically depicted. Reprinted with permission from ref (150). Copyright 2019 Springer Nature.

The two main forms of sensitizing agents, N-doped graphene quantum dots (N-GQDs-NSs) coupled to nanosheets and N-doped carbon nanoparticles (N-CNPs) (Figure 9), have been produced from natural carbon powder utilizing the straightforward methods of oxidation and centrifuge separation. Analysis using the techniques of XRD, UV–vis, FT-IR, FE-SEM, XPS, Raman spectroscopy, AFM, HR-TEM, and FL revealed that nitrogen had been successfully incorporated into carbon nanoparticles, providing them an average plane dimension of 50 nm and a reasonably smooth surface. The capacity of the produced samples to identify volatile organic substances using a simple optical-fiber-based sensor setup was used to create and establish the samples’ adaptability for sensitizing agents. Comparative laboratory studies have explored that the recommended sensor’s efficiency can respond quickly within just a few tens of seconds when exposed to methanol vapor. When exposed to various alcohol vapors in an atmosphere with ambient air, their lower limits of detection were, respectively, 4.3, 4.9, and 10.5 ppm.^150^

**Figure 10:**
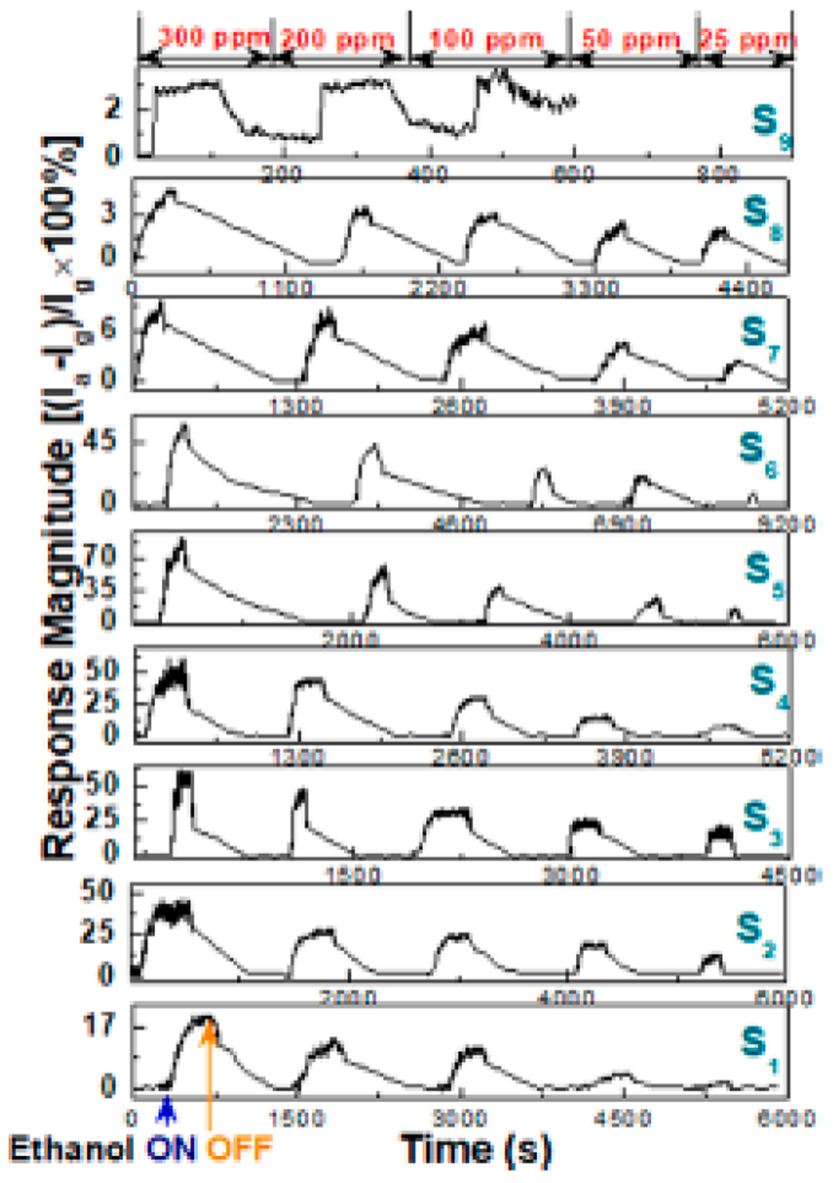
Transient behavior of S1–S9 sensors in the 25–300 ppm range of ethanol concentration at 100 °C. VGS = 0 and VDS = 0.5. Reprinted with permission from ref (151). Copyright 2021 Elsevier.

For rapid identification of VOCs, field effect transistors (FETs) with a p-TiO_2_ nanoparticle (NPs)/GO heterojunction are used. P-type anatase TiO_2_ NPs made from sol–gel were used as the channel materials and were implanted in a few stacked GO channels. To determine the ideal ratio of GO and TiO_2_ NPs within the hybrid channel, extensive microscopic, spectroscopic, and electrical characterizations were carried out. On a SiO_2_/Si substrate, a back-gated FET sensor was created and put through testing while being exposed to various VOCs. At 100 °C, tests with p-TiO_2_/GO FET sensors at zero gate voltage (VGS = 0) demonstrated ethanol selectivity. The lower detection limit of ethanol was enhanced to 500 ppb by utilizing an appropriate gate voltage (VGS > 0, near the Dirac point), significantly enhancing its sensitivity and p-TiO_2_–GO hybrid for field-assisted sensitivity amplification. Transient behavior in the presence of reducing vapor ethanol, on the other hand, served as evidence for the p-type conductance of the composites of p-TiO2 NPs and p-GO. The pure GO sensor (S9) could not respond at 100 ppm, but the S1–S8 sensors produced a response within the dynamic range of 25–300 ppm. Every sensor demonstrated a stable baseline with predictable sensing behavior (Figure 10).^151^

**Figure 11:**
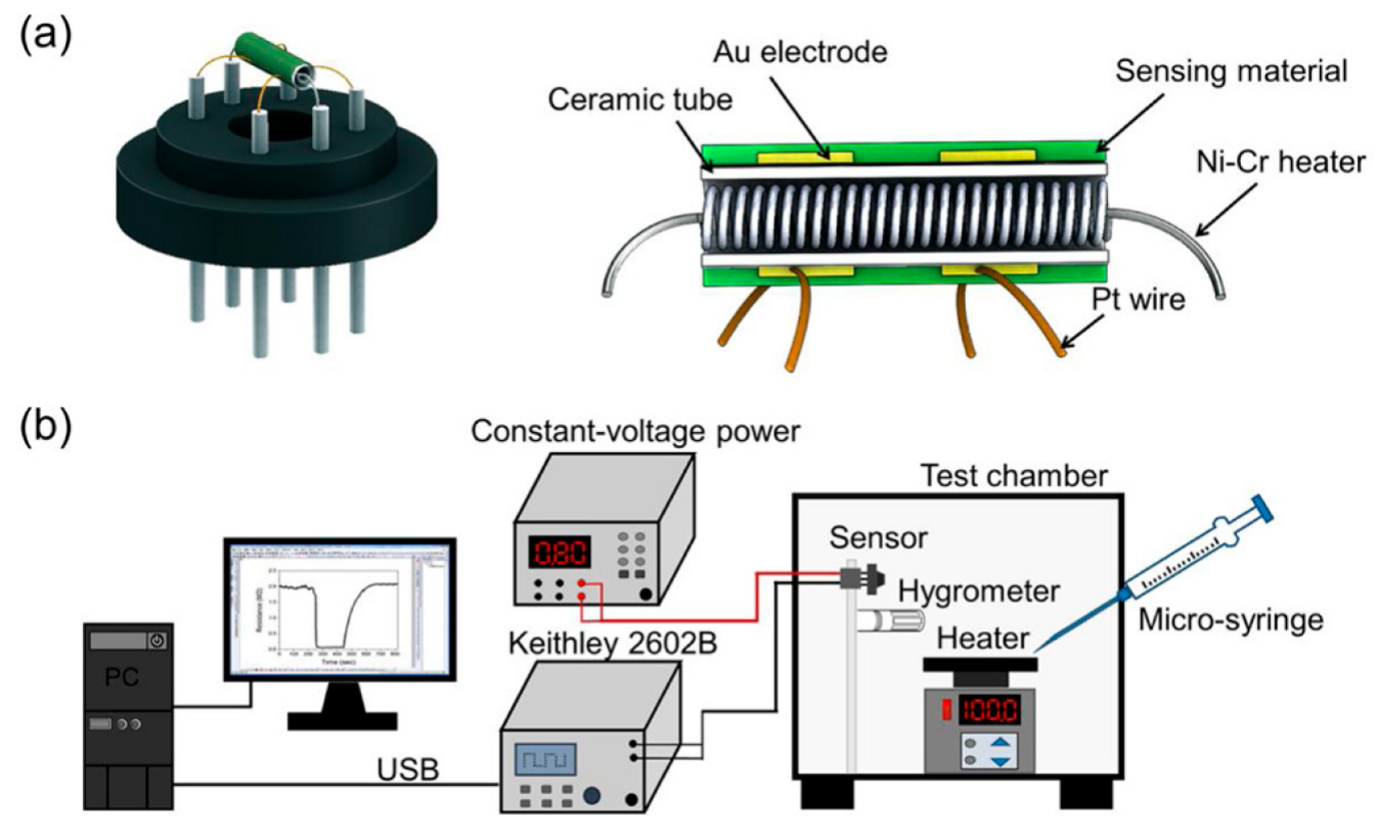
Schematic structure of a whole gas sensor in (a) and the test and analysis system in (b). Reprinted with permission from ref (152). Copyright 2020 Elsevier.

A susceptible HCHO sensor is suggested and demonstrated using a heterostructure of the Sn_3_O_4_/rGO composite. It features a low working temperature and a wide detection range. The hydrothermal Sn_3_O_4_/rGO composite exhibits a large specific surface area and a microstructure like a flower. The sensing features of sensors based on pure Sn_3_O_4_ and Sn_3_O_4_/rGO composites are thoroughly analyzed to learn how the heterostructure influences the sensing performance of HCHO. According to the observed data, the Sn_3_O_4_/rGO composite sensor has a significantly higher sensing response at a lower working temperature than the pure Sn_3_O_4_ sensor. Figure 11a,b presents data analysis and the schematic configuration of the gas sensor.^152^

As one of the most frequently harmful irritating gases, NO_2_ can cause asthma and other illnesses in people.^153^ Researchers are concentrating on creating light, affordable wearable electronics with excellent performance, and adaptability due to the recent rapid advancement of gas sensor research.^154^ Chen and a colleague created a smartphone-enabled, completely integrated wireless system for real-time NO_2_ monitoring using a flexible ZnS nanoparticle/nitrogen-doped reduced graphene oxide (ZnS NP/N-rGO) sensor. The device exhibited a response of 2.2–10 ppm of NO_2_, a rapid recovery performance of 724 s, a power consumption of 0.52 W, and an extremely low theoretical limit of detection (69 ppb) for graphene-based sensors.^155^

## 8 CD-Based Sensor for VOC Detection

**Figure 12:**
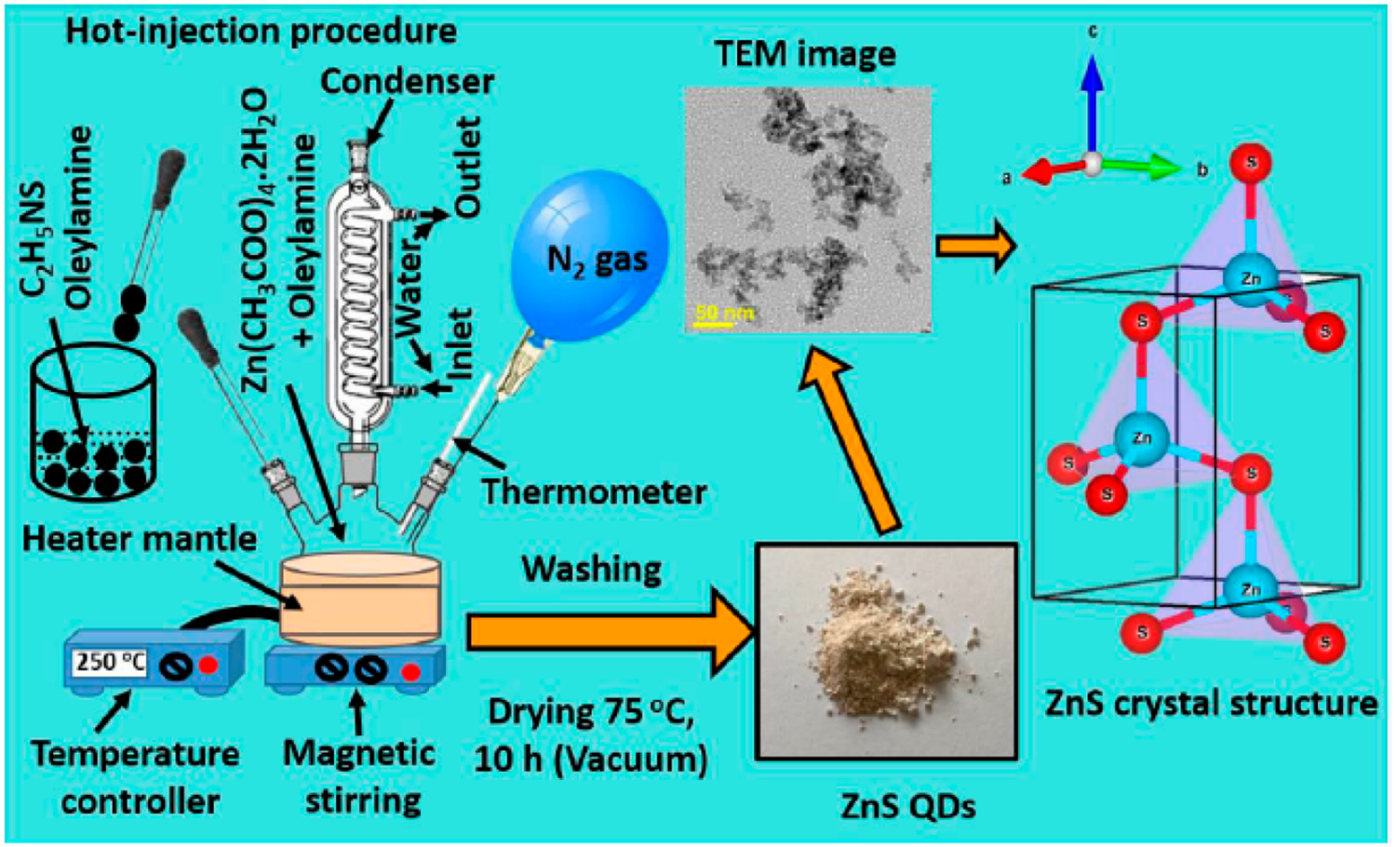
Schematic picture of the hot-injection process used to create ZnS quantum dots (QDs) and an illustration of the ZnS crystal structure. Reprinted with permission from ref (157). Copyright 2021 MDPI.

For their excellent environmentally friendly nature, brightness, tunable fluorescence, inertness, low cost, straightforward synthetic route, and availability for a wide range of starting materials, carbon dots (Cdots), an emerging nanomaterial of the carbon-based materials family, have attracted significant research interest.^156^ For use in acetone gas-sensing applications, the Mishra research group reports producing ZnS quantum dots (QDs) via a hot-injection technique (Figure 12). The generated ZnS QDs were characterized using XRD and TEM analysis. The ZnS QD sensor exhibits rapid response and recovery times. At a 100 ppm acetone concentration at 175 °C, it also demonstrates outstanding stability, high sensitivity, and strong selectivity. Table 2 also compares the ZnS nanomaterial-based acetone sensor with earlier works or research tools based on metal-oxide nanoparticles. The ZnS QD sensor can be a viable sensor to detect acetone vapor from exhaled air for the invasive type 1 diabetes inspection.^157^

**Table 2:** Comparative Study of ZnS-Based Nanosensors with Metal Oxide Nanosensors for Acetone Sensing

ZnS-based sensor	*T*/°C	acetone (ppm)	response time (s)	recovery time (s)	detection limit	stability/selectivity	ref
ZnS QDs	175	100	5.5	6.7s	12 ppm	89.1%/91.1%	(157)
ZnS nanowire	320	100	∼30	∼45	–	–/21.1	(158)
Au/ZnS	260	100	–	–	–	–/–	(159)
ZnO@ZnS core/shell	300	500	∼10	∼16	–	cycling for 55 s/-	(160)
Cr_2_O_3_/ZnS	300	200	–	–	–	–/–	(161)
20Nb/WO_3_	325	1	∼7	∼625	–	∼14.3/22.3	(162)
TiO_2_/α-Fe_2_O_3_	300	300	∼22	∼86	–	∼23/23	(163)
NiO/Zn_2_SnO_4_	300	100	∼1	∼60	<100 ppb	∼5 cycles for 1000 s/49.6	(164)
SnO_2_/ZnSnO_3_	290	300	∼5	∼115	–	∼31/32	(165)

**Figure 13:**
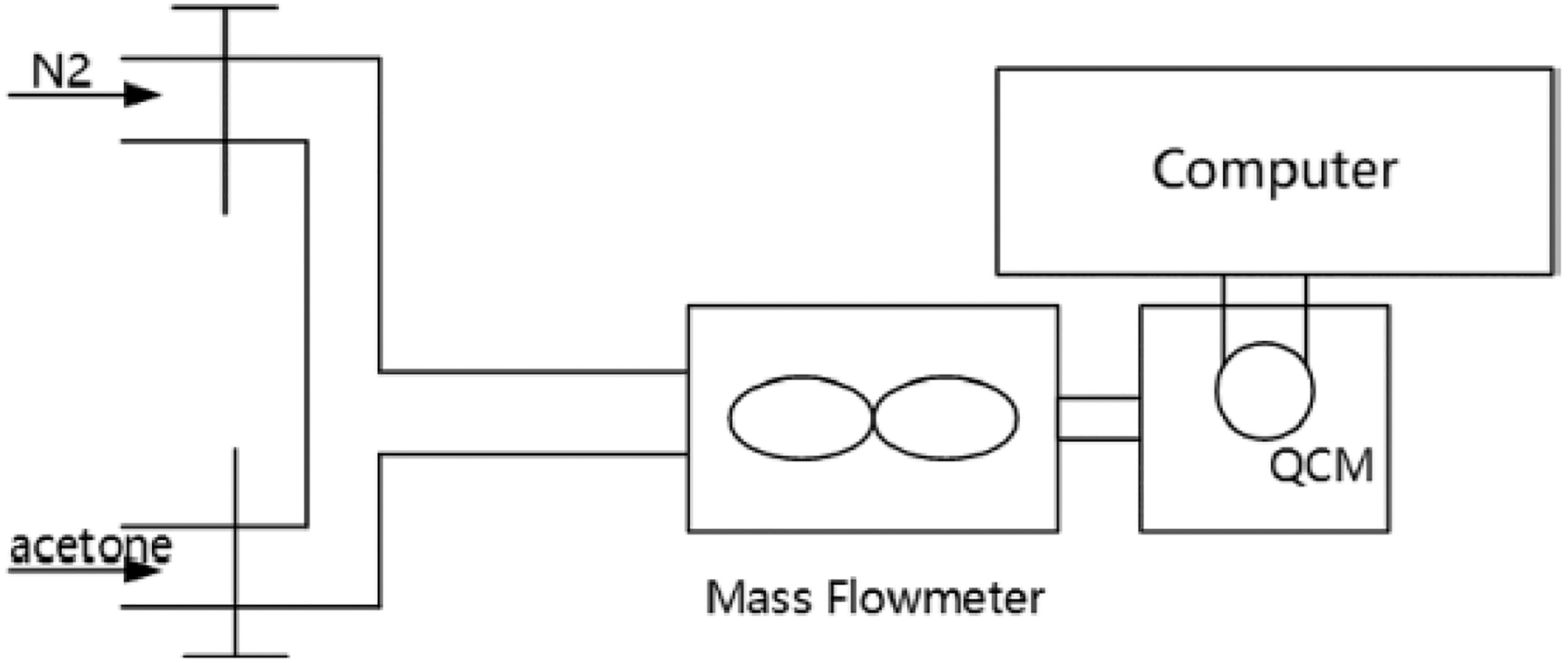
Schematic of VOC detection device. Reprinted with permission from ref (166). Copyright 2021.

An acetone sensor was created using a quartz crystal microbalance (QCM), which was modified with graphene quantum dots (GQDs) to sense gases. GQDs were produced via citrate pyrolysis, and their characteristics were determined by high-resolution TEM (HR-TEM). They looked at the sensor’s gas sensitivity to acetone at low concentrations. With a sensitivity of 16.78 Hz/ppm and a minimal detection limit of 2.5 ppm, it demonstrated good linearity at less than 240 ppm acetone concentrations. The sensor showed high acetone selectivity in a combination of acetone, butanol, and isopropanol (Figure 13). The same sensor’s response time was constant for varying acetone concentrations, and the response and recovery durations were 32 and 48 s, respectively.^166^

## 9 Other Carbon Nanomaterial-Based Sensors for the Detection of VOCs

The gas-sensing properties of MXene may be predicted using the quantum mechanical approach and basic principles based on density functional theory (DFT). Using the Schrödinger equation, DFT-based first-principles examine the electronic structure of materials.^167^ The absorption energy of material to certain gas molecules, as well as the associated charge transfer characteristics, are important factors in predicting sensing qualities based on gas sensing. Yu et al. performed the first computational analysis to demonstrate MXenes’ potential as a gas sensor or capturer, employing monolayer Ti_2_CO_2_ for NH_3_ detection.^168^ Because metallic Ti_2_C(OH)_2_, Ti_2_CF_2_, and Ti_2_C do not have semiconducting properties, only Ti_2_CO_2_ was studied using first-principles modeling. Using the most stable structure, the O-functionalized monolayer of Ti_2_CO_2_, gas molecules’ possible absorption positions were investigated to compute adsorption energy and charge transfer. Aside from demonstrating gas absorption and charge transfer in MXenes, plane-wave-based DFT computation allows for the tailoring of MXene selectivity via controlled oxygen functionalization. Junkaew et al. investigated gas molecule adsorption behavior on four MXenes (M_2_C, M = Ti, V, Nb, Mo) and their O-terminated surfaces with electronic charge characteristics.^168^ When a gas molecule is absorbed, it retains its molecule form or is dissociated on the surface of the MXenes. Theoretical simulations predicted varied absorption energies of multiple gaseous species on various types of MXene surface conditions and their associated charge transfer for gas-sensing properties. However, just a few laboratory experiments on the sensing properties of different MXenes have been published. We report a novel experimental investigation into the gas-sensing properties of MXenes. Layered transition metal carbides, nitrides, or MXene are composed of more than 60 different types, including Ti_2_C, V_2_C, Nb_2_C, Ti_4_N_3_, TiNbC, Ti_3_CN, and Mo_2_TiC_2_.^169−171^ By chemically treating precursors of the M_*n*+1_AX_*n*_ phase, also known as the MAX phase, where M is an early transition metal, A is typically an element IIIA or IVA; X is C and/or N; and *n* = 1, 2, and 3 MXenes are produced.^172^ MXenes are a focus of research because of a number of intriguing characteristics that promise to lead the way for future sensor development. A fascinating class of two-dimensional materials called MXenes has lately attracted interest for their possible use in gas sensing.^173^

Here, we show the efficiency of a new Mo_2_CT_*x*_ MXene sensor in detecting VOCs. On a Si/SiO_2_ substrate, the suggested sensor is a chemiresistive device made by photolithography. The effectiveness of the sensor is examined in relation to different MXene manufacturing conditions. Different VOC concentrations, including ethanol, toluene, benzene, acetone, and methanol, are examined at room temperature. The effectiveness of several MXene-based gas sensors is compared in Table 3.^174^

**Table 3:** Comparison of MXene-Based Gas Sensors at Room Temperature (RT)

MXene sensor	operating temperature	VOCs with highest sensing response	LOD	cross-sensitivity ratio (at 100 ppm)	ref
Mo_2_CT_*x*_	RT	toluene	220 ppb	2.65	(175)
Ti_3_C_2_T_*x*_	RT	NH_3_	–	1.47	(176)
Ti_3_C_2_T_*x*_	RT	C_2_H_5_OH	50 ppb	1.75	(177)
V_2_ C_2_T_*x*_	RT	C_2_H_5_OH	–	3.61	(178)
CuO/Ti_3_C_2_T_*x*_	250 °C	toluene	320 ppb	–	(179)
S-doped Ti_3_C_2_T_*x*_	RT	toluene	–	–	(180)

**Figure 14:**
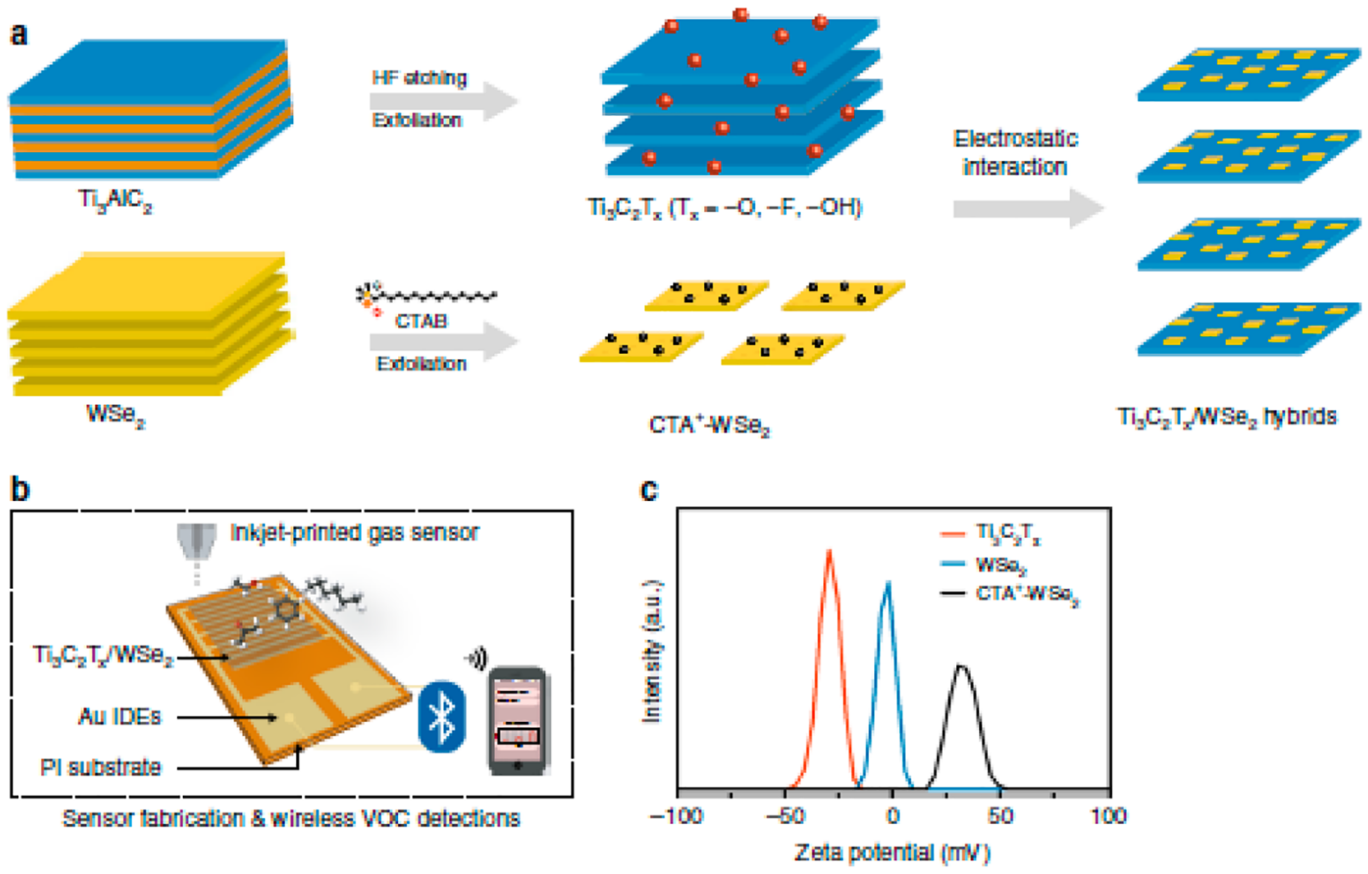
(a) Ti_3_C_2_T_*x*_/WSe_2_ hybridization and sensor construction. Ti_3_C_2_T_*x*_/WSe_2_ nanohybrid preparation procedures are shown schematically. (b) Schematic representation of inkjet-printed gas sensors used in a wireless monitoring system to detect volatile organic chemicals: WSe_2_, CTA + −WSe_2_, and Ti_3_C_2_T_*x*_ dispersions. (c) Zeta potential distributions. Reprinted with permission from ref (173). Copyright 2020 Springer Nature.

In this case, the model materials for hybridization and their application to the detection of different volatile organic chemicals are Ti_3_C_2_T_*x*_ and WSe_2_ (Figure 14). The Ti_3_C_2_T_*x*_/WSe_2_ hybrid sensor has excellent adaptability for a wide range of volatile organic compounds, low noise levels, and exceptionally fast response and recovery times. The hybrid sensor’s sensitivities to ethanol have risen about 12-fold when compared to pure Ti_3_C_2_T_*x*_. Furthermore, by restricting the contact of water molecules from Ti_3_C_2_T_*x*_’s edges, the hybridization process offers a successful defense against MXene oxidation. Detecting volatile organic molecules containing oxygen is highly sensitive and selective, and an enhanced method for Ti_3_C_2_T_*x*_/WSe_2_ heterostructured materials is presented.^173^

It is highlighted how well the Zhao research team’s novel MXene, V_4_C_3_T_*x*_, functions as an acetone sensor. It was made from V_4_AlC_3_ by selectively etching the Al layer with aqueous HF RT. A V_4_C_3_T_*x*_-based acetone sensor operates well due to its low operating temperature of 25 °C, low detection limit of 1 ppm below the 1.8 ppm diabetes diagnostic threshold, and good selectivity toward acetone in a mixed mixture of water vapor and acetone. It might lead to a much faster and earlier diagnosis of diabetes. V_4_C_3_T_*x*_ MXene is used for the first time in this work in the context of acetone detection.^180^

We provide a simple solvothermal method for fabricating W_18_O_49_/Ti_3_C_2_T_*x*_ composites that build 1D W_18_O_49_ nanorods (NRs) upon the surfaces of 2D Ti_3_C_2_T_*x*_ Mxene sheets. The 1D W_18_O_49_ NRs were uniformly distributed throughout the surfaces of a 2D Ti_3_C_2_T_*x*_ sheet to produce the 1D/2D W_18_O_49_/Ti_3_C_2_T_*x*_ hybrids. Comparing the W_18_O_49_/Ti_3_C_2_T_*x*_ composites to the Ti_3_C_2_T_*x*_ sheets and W_18_O_49_ NRs, it was discovered that they markedly increased the ability to sense acetone. The hybrid sensor demonstrated a robust response to low acetone concentrations, rapid response and recovery times, excellent selectivity, long-term stability, and an extremely low acetone detection limit. The superb sensing performance of the hybrid sensor can be attributed to the uniform dispersion of W_18_O_49_ NRs on the surface, the elimination of fluorine-containing groups during the solvothermal process, and the beneficial interactions at the interface between the W_18_O_49_/Ti_3_C_2_T_*x*_ sheets and W_18_O_49_ NRs.^181^

Schottky-barrier-equipped Ti_3_C_2_T_*x*_–ZnO nanosheet hybrids have been produced for NO_2_ recovery and detection under UV light. By using HF solution to etch the Ti_3_AlC_2_ Al layer, Ti_3_C_2_T_*x*_ nanosheets were produced (MAX). ZnO nanosheets’ porous structure is crucial for the adsorption of NO_2_ gas. With the aid of UV irradiation during the recovery process, the Ti_3_C_2_T_*x*_–ZnO nanosheet-based gas sensor displayed better NO_2_-detecting skills, including a high sensitivity of 367.63% to 20 ppm of NO_2_ and a quicker response/recovery time of 22 s/10 s.^182^

**Figure 15:**
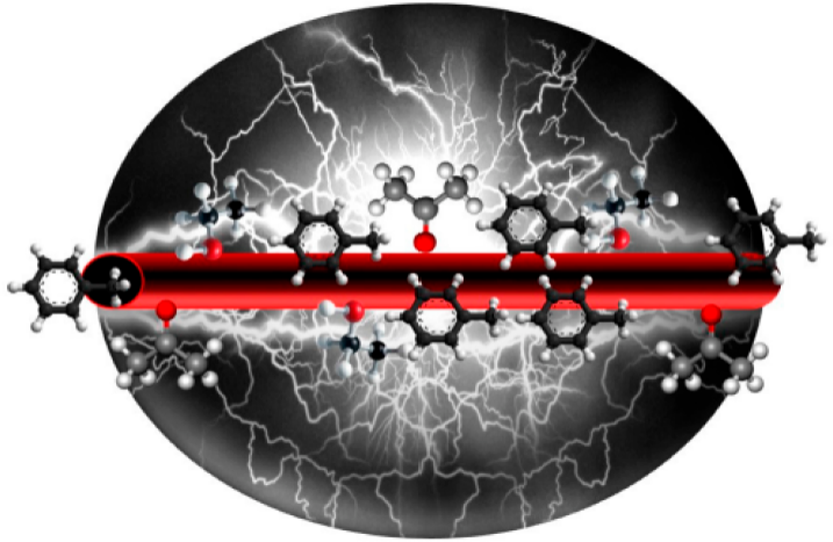
Electrically conductive oxygenated amorphous carbon (a-CO*_x_*)/graphite (G) (a-CO*_x_*/G) nanofibers’ ability for sensing VOCs. Reprinted with permission from ref (183). Copyright 2019 MDPI.

The first oxygenated amorphous carbon (a-CO_*x*_)/graphite (G) nanofilament-based buckypaper sensor was developed by Homaeigohar (Figure 15). By forming hydrogen bonds, oxygen-containing groups influence the group’s capacity to extract electrons, the density of hole carriers, and, therefore, the resistivity, which in turn affect the group’s selectivity toward VOCs like ethanol and acetone. However, the creation of charge-transfer complexes caused by the toluene aromatic ring’s electrostatic interactions with the electrons of the graphitic crystals may be the main cause of the sensor’s great responsiveness to nonpolar toluene.^183^

## 10 Conclusions, Outlook, and Perspective

The development of VOC sensing utilizing CNMs is outlined in this comprehensive review. Due to the wide range of uses for VOC environmental monitoring, the difficulty of selecting VOC detection is growing every day. Toxic VOC detection is crucial for protecting the environment and human health. Due to their unique morphology and features, developing CNMs has been the foundation for sensor technology over the preceding two decades. CNMs like graphene, CNTs, and their derivatives, which have a large number of adsorption sites, low density, tunable electrical properties, high carrier mobility, low operating temperatures, longer lives, and ease of recovery, are appropriate sensing materials for a variety of toxic pollutant gases and volatile organic compounds. Numerous researchers have observed an increase in sensitivity that allows for detecting harmful VOCs at ppb levels. The selectivity of a CNM-based sensor toward a specific VOC at room temperature has been shown in numerous papers. According to the literature, metal oxide and nanocarbon hybrids have outstanding sensing capabilities, with nanocarbon serving as the primary component. Composites made of nanocarbon have shown a strong potential for use in VOC sensing.

However, some measure of research should be focused on the synthesis or design of porous or hierarchical structures of carbon-based materials for the suitable VOC molecule adsorption with more reaction sites which can help with a better understanding of structural-adsorption/absorption property relationships that will garnish sensing mechanisms. Also, by analysis of theoretical calculation, the change of electronic properties of host–guest items can be explored for selective gas sensors.
